# Determinants of Group B streptococcal virulence potential amongst vaginal clinical isolates from pregnant women

**DOI:** 10.1371/journal.pone.0226699

**Published:** 2019-12-18

**Authors:** Lindsey R. Burcham, Brady L. Spencer, Lauryn R. Keeler, Donna L. Runft, Kathryn A. Patras, Melody N. Neely, Kelly S. Doran

**Affiliations:** 1 Department of Immunology and Microbiology, University of Colorado, Aurora, CO, United States of America; 2 Department of Biology, San Diego State University, San Diego, CA, United States of America; 3 Department of Molecular & Biomedical Sciences, University of Maine, Orono, ME, United States of America; Cairo University, EGYPT

## Abstract

*Streptococcus agalactiae*, also known as Group B *Streptococcus* (GBS), is a Gram-positive bacterium isolated from the vaginal tract of approximately 25% of women. GBS colonization of the female reproductive tract is of particular concern during pregnancy as the bacteria can invade gestational tissues or be transmitted to the newborn during passage through the birth canal. Infection of the neonate can result in life-threatening pneumonia, sepsis and meningitis. Thus, surveillance of GBS strains and corresponding virulence potential during colonization is warranted. Here we describe a panel of GBS isolates from the vaginal tracts of a cohort of pregnant women in Michigan, USA. We determined that capsular serotypes III and V were the most abundant across the strain panel, with only one isolate belonging to serotype IV. Further, 12.8% of strains belonged to the hyper-virulent serotype III, sequence type 17 (ST-17) and 15.4% expressed the serine rich repeat glycoprotein-encoding gene *srr2*. Functional assessment of the colonizing isolates revealed that almost all strains exhibited some level of β-hemolytic activity and that ST-17 strains, which express Srr2, exhibited increased bacterial adherence to vaginal epithelium. Finally, analysis of strain antibiotic susceptibility revealed the presence of antibiotic resistance to penicillin (15.4%), clindamycin (30.8%), erythromycin (43.6%), vancomycin (30.8%), and tetracycline (94.9%), which has significant implications for treatment options. Collectively, these data provide important information on vaginal GBS carriage isolate virulence potential and highlight the value of continued surveillance.

## Introduction

GBS is commonly isolated from the lower gastrointestinal tract or the female reproductive tract. Though normally an asymptomatic colonizer in these environments, this opportunistic pathogen can cause invasive disease, including pneumonia, sepsis, and meningitis [1]. The newborn is one of the primary populations at risk for the development of GBS invasive disease, with infection occurring through vertical transmission from the mother either by ascending bacterial infection *in utero* or during passage of the fetus through the colonized birth canal. Neonatal invasive disease is classified into two distinct categories: early-onset disease (EoD), or late-onset disease (LoD) [2, 3]. Early-onset infections typically occur in the first week of life, presenting acutely with pneumonia and respiratory failure complicated by bloodstream infection, septicemia, and sometimes meningitis. In contrast, GBS LoD occurs in infants up to 7 months of age, with more indolent symptom progression related to bacteremia and a high incidence (~50%) of meningitis [4]. As a result of the high risk of infection in the neonate, implementation of late gestational screening and prescription of prophylactic antibiotics have become common practice in the US for expectant mothers during late stages of pregnancy or at delivery [5]. Colonized women are typically administered penicillin as a first-line drug, but in the case of allergy or suspected resistance, are instead treated with clindamycin, erythromycin or in some cases vancomycin [1]. Despite the initial effectiveness of the antibiotic treatment in decreasing GBS EoD in 2002, the rate of overall EoD increased during 2003–2005, reflecting increases in incidence among Black infants [6]. The Center for Disease Control reports that as of 2017, GBS remains a leading cause of neonatal sepsis and meningitis, with a national estimate approaching 1000 live births annually (0.22/1000) in the United States developing early-onset meningitis [7].

GBS possesses an arsenal of virulence factors, which contribute to host cell attachment, invasion, colonization and progression of invasive disease [8]. The sialylated GBS capsular polysaccharide (CPS) represents one of the most critical virulence factors and, thus far, ten capsular serotypes have been identified (Ia, Ib and II–IX). Of the 10 different GBS serotypes described, Ia, Ib, II, III, and V are more commonly associated with disease and account for the majority of cases worldwide [9]. GBS has also been classified by sequence type (ST) based on an allelic profile of seven different loci [10]. Serotype III strains belonging to the ST-17 background represent a hyper-virulent lineage, which causes a disproportionately high incidence of neonatal invasive disease and meningitis [11]. Additional well-studied GBS virulence factors include the β-hemolysin/cytolysin (β-H/C) [12–14], serine-rich repeat surface glycoproteins (Srr1/2) [15–19], pilus proteins [20–24], and surface adhesins known to interact with extracellular matrix components [18, 25]. However, the contribution of these virulence factors to GBS colonization of the vaginal tract is still being elucidated.

In this study, we characterized a panel of 39 GBS clinical isolates collected from the vaginal tracts of pregnant women at the Detroit Medical Center in Detroit, Michigan [19]. In order to characterize the disease potential of our strain panel, we assessed capsular serotype by PCR or flow cytometry and the hyper-virulent sequence type (ST-17) in serotype III strains. We also assessed virulence potential by determining vaginal cell adherence, hemolysis, and susceptibility against five of the most clinically relevant antibiotics within the U.S. health care system: penicillin, clindamycin, erythromycin, vancomycin, and tetracycline.

## Materials and methods

### Bacterial strains and cell lines

*Streptococcus agalactiae* (GBS) clinical isolate strains used in this study were A909 [26], CJB111(ATCC BAA-23) [27], CNCTC10/84 [28], COH1 [29], H36B [30] and the β-H/C-deficient COH1Δ*cylE* [13]. GBS strains isolated from the vaginal tracts of pregnant women were purchased from the Detroit Medical Center, Detroit, Michigan; thus, no patient history information is known. Strains were grown on Chrome indicator Agar for GBS (CHROMagar StrepB SB282) as well as confirmed by latex test to confirm the presence of the Group B carbohydrate (Remel Streptex R30950701) [19]. Immortalized human vaginal epithelial cells (VK2/E6E7, ATCC CRL-2616) were maintained in keratinocyte serum-free media (Life Technologies) with the addition of 0.1 ng/mL human recombinant epidermal growth factor and 0.05 mg/mL bovine pituitary extract at 37°C in 5% CO_2_ as described previously (18).

### Serotyping by PCR and flow cytometric serotyping assay (FCSA)

Molecular serotyping was performed by PCR amplification using Taq DNA polymerase (New England Biolabs, Massachusetts, USA) and primer sequences previously described by Poyart et al. [31] (Table 1). Because the serotype III primer set has potential to cross-react with serotype Ia and II *cps* loci due to high sequence identity [31], these serotypes were confirmed by flow cytometry using monoclonal antibodies specific for capsular serotypes Ia, II, or III. Since an anti-Ib monoclonal antibody is also available, serotype Ib isolates were further confirmed by flow cytometry, as described previously [32], with slight modifications. Monoclonal antibodies specific to GBS serotypes Ia, Ib, II, and III were provided by John Kearney at the University of Alabama at Birmingham [33–36]. Serotypes IV and V were determined by PCR alone as monoclonal antibodies specific to serotypes IV and V are not available. Briefly, bacteria were grown to OD_600nm_ = 0.4 and then normalized by to yield ~5x10^5^ CFU/ well in HBC buffer (1× HBSS without magnesium or calcium, 0.5% Bovine serum albumin, 2.2 mM CaCl_2_). Monoclonal antibodies to serotypes Ia, Ib, II, and III were incubated with bacteria at predetermined concentrations of 48 (Ia), 89 (Ib), 6.6 (II), and 77 ng/μL (III) respectively for 30 minutes at 4°C with shaking. Wells were washed two times with HBC and bacteria were incubated with donkey anti-mouse IgM-AF647 (1:2000 final dilution) 30 minutes at 4°C with shaking. Bacteria were washed two times, resuspended in HBC, and analyzed by flow cytometry on a FACSCalibur flow cytometer. Flow cytometric data was analyzed by FlowJo (V10).

**Table 1:** Primer sequences.

Primer Name	Sequence 5’ → 3’
Serotype Ia Forward	GGTCAGACTGGATTAATGGTATGC
Serotype Ia Reverse	GTAGAAATAGCCTATATACGTTGAATGC
Serotype Ib Forward	TAAACGAGAATGGAATATCACAAACC
Serotype Ib Reverse	GAATTAACTTCAATCCCTAAACAATATCG
Serotype II Forward	GCTTCAGTAAGTATTGTAAGACGATAG
Serotype II Reverse	TTCTCTAGGAAATCAAATAATTCTATAGGG
Serotype III Forward	TCCGTACTACAACAGACTCATCC
Serotype III Reverse	AGTAACCGTCCATACATTCTATAAGC
Serotype IV Forward	GGTGGTAATCCTAAGAGTGAACTGT
Serotype IV Reverse	CCTCCCCAATTTCGTCCATAATGGT
Serotype V Forward	GAGGCCAATCAGTTGCACGTAA
Serotype V Reverse	AACCTTCTCCTTCACACTAATCCT
*hvgA* Forward	ATACAAATTCTGCTGACTACCG
*hvgA* Reverse	TTAAATCCTTCCTGACCATTCC
*srr1* Forward	AGTGTCTGATACTGAAATGTTAGGTA
*srr1* Reverse	TAGATTCCCAAGTCCTGATGCGA
*srr2* Forward	GTTCCGAGTCAGTATCAATGAG
*srr2* Reverse	AATCCTGCACCTAACAGACC

### Determination of hemolytic activity

To determine β-hemolytic activity, strains were grown overnight at 37°C and 10 μL was spotted onto sheep blood agar plates (Remel R01200) and incubated overnight at 37°C with 5% CO_2_ [37]. The hemolytic activities of vaginal isolates were compared to reference strains COH1Δ*cylE* (-), COH1 (+), A909 (++), and CNCTC10/84 (+++) via blind scoring by two individuals for three biological replicates. Phenotypes from overnight cultures were confirmed using cultures grown to OD_600nm_ 0.4.

### Molecular identification of factors contributing to colonization

Isolates were confirmed by PCR for the presence of the genes encoding serine-rich repeat glycoproteins 1 and 2 (*ssr1*, *srr2*) [19]. Additionally, all serotype III vaginal isolates were assessed for hyper-virulent sequence type 17 (ST-17) lineage based on detection of the *hvgA* gene (*gbs2018C*) [38]. Primer sequences used throughout this study are listed in Table 1.

### Bacterial adherence assay

GBS adherence to VK2/E6E7 was determined as described previously [17, 19, 39, 40]. Briefly, vaginal epithelial cells were seeded into tissue culture treated 96-well plates and grown to 90–100% confluence. Clinical isolates were grown to OD_600nm_ 0.4 in Todd-Hewitt broth and inoculated onto vaginal cell monolayers at 1x10^4^ CFU, MOI = 1. Plates were centrifuged at 1000 RPM (169 x g) for 5 minutes and incubated at 37°C with 5% CO_2_ for 30 minutes. Following incubation, plates were rinsed five times with PBS to remove non-adherent bacteria. Vaginal cells were then detached with 0.25% trypsin-EDTA and lysed with 0.025% Triton-X-100 in phosphate-buffered saline. Lysates were diluted and plated to quantitate adherent CFU. Assays were performed in triplicate wells in biological triplicate experiments.

### Antibiotic sensitivity profiling

Overnight cultures of GBS strains were spread on Todd Hewitt agar. Discs impregnated with antibiotics at the following concentrations were added and plates were incubated overnight at 37°C (Penicillin 10 U, Clindamycin 2 mg, Erythromycin 15 mg, Vancomycin 30 μg, and Tetracycline 30 μg) [41]. Following overnight incubation, zones of inhibition surrounding antibiotic discs were measured. Disc diffusion was performed two times for each strain and antibiotic tested. Zones of inhibition were characterized as susceptible, intermediate, or resistant based on the following measurement determinations: Penicillin (Resistant ≤23 mm, Susceptible ≥24 mm), Clindamycin (Resistant ≤15 mm, Intermediate 16–18 mm, Susceptible ≥19 mm), Erythromycin (Resistant ≤15 mm, Intermediate 16–20 mm, Susceptible ≥21 mm), Vancomycin (Resistant ≤16 mm Susceptible ≥17 mm), and Tetracycline (Resistant ≤18 mm, Intermediate 19–22, and Susceptible ≥23 mm). As penicillin and vancomycin do not have an intermediate range, zones measuring larger than the susceptible limit were deemed resistant.

### Statistical analyses

Adherence assays performed in this study were conducted three times independently with triplicate samples. Data are displayed as averages from the three independent experiments and analyzed by one-way ANOVA with Sidak’s multiple comparisons. All analyses were conducted with α = 0.05 and statistical significance determined as *p* < α. All statistical analyses were performed using GraphPad Prism 7 and R Studio.

## Results

### Serotype distribution and virulence potential of vaginal clinical isolates

Molecular serotype was determined by PCR using primers specific to various serotypes’ *cps* loci as described in Poyart et al. [31] (Table 2). Serotypes Ia, Ib, II, and III were confirmed by flow cytometry, utilizing monoclonal antibodies against GBS capsule (S1 Fig). Serotypes V and III constituted over half of the colonizing isolates at 28.2% (11/39) and 25.6% (10/39), respectively (Table 2). The remaining isolates were identified as serotype II (15.4%, 6/39), Ia (12.8%, 5/39), Ib (15.4%, 6/39), and IV (2.6%, 1/39) (Table 2). In addition to capsular serotype, GBS strain background is also known to contribute to virulence potential. In particular, strains belonging to sequence type 17 (ST-17) are hyper-virulent and commonly associated with invasive disease, specifically meningitis [10, 38]. Additional bacterial factors known to contribute to virulence in GBS are the serine-rich repeat glycoproteins Srr1 or Srr2 [18, 19, 42, 43]. We therefore assessed the presence of the *hvgA* gene (*gbs2018C*) (indicative of ST-17) and *srr1*/*srr2* genes within our strain panel by PCR. We confirmed that 50% of our serotype III strains belonged to the ST-17 lineage and encoded *srr2* (Table 2). The sole serotype IV strain within our panel also encoded *srr2* whereas the remaining 31/33 (94%) GBS vaginal isolates encoded *srr1*.

**Table 2:** Clinical isolate serotype distribution.

Serotype	Ia	Ib	II	III	IV	V
Distribution	5/39 (12.8%)	6/39 (15.4%)	6/39 (15.4%)	10/39 (25.6%)	1/39 (2.6%)	11/39 (28.2%)
**Strain numbers**	5^**◊**^ 17^**◊**^ 48^**◊**^ 67^**◊**^ 68^**◊**^	3^**◊**^ 10^**◊**^ 14^**◊**^ 30^**◊**^ 62^**◊**^ 63^**◊**^	6^**◊**^ 16^**◊**^ 31^**◊**^ 54^**◊**^ 56^**◊**^ 64^**◊**^	4*^#^ 9*^#^ 24*^#^ 29 40*^#^ 43^**◊**^ 52 59^**◊**^ 69*^#^ 71^**◊**^	51^#^	1^**◊**^ 2^**◊**^ 12^**◊**^ 15^**◊**^ 20^**◊**^ 27^**◊**^ 28^**◊**^ 33^**◊**^ 39^**◊**^ 41^**◊**^ 57^**◊**^

The displayed symbols correlate with isolates of sequence type-17 (ST-17) (*) or isolates expressing *srr1* (◊) or *srr2* (#).

### Hemolytic activity

GBS hemolytic activity is associated with increased virulence [12–14]; thus, we assessed hemolytic activity of the vaginal clinical isolates as described in the Methods. We compared the observed hemolytic activity of GBS vaginal isolates to reference GBS strains COH1Δ*cylE* (-), COH1 (+), A909 (++), and CNCTC10/84 (+++). The majority of vaginal isolates exhibited hemolysis at levels similar to invasive isolates COH1 (19/39, 48.7%) and A909 (16/39, 41%) (Table 3, S2 Fig). However, we also identified two non-hemolytic isolates (similar to the COH1Δ*cylE* strain) and two hyper-hemolytic isolates (similar to the CNCTC10/84 strain). Collectively, a wide range of hemolytic activity was observed across our isolate panel, with most strains displaying detectable but low hemolytic activity. (Table 3, S2 Fig)

**Table 3:** Clinical isolate hemolytic activity.

Clinical Isolate Hemolytic Activity				
	-	+	++	+++
Reference Strain	COH1Δ*cylE*	COH1	A909	NCTC 10/84
Vaginal Isolates	29, 52	1, 2, 4, 12, 16, 17, 24, 28, 31, 33, 39, 40, 41, 51, 54, 56, 57, 64, 69	3, 5, 6, 9, 14, 15, 20, 27, 30, 43, 59, 62, 63, 67, 68, 71	10, 48

### Adherence of clinical isolates to vaginal epithelial cells

**Fig 1:**
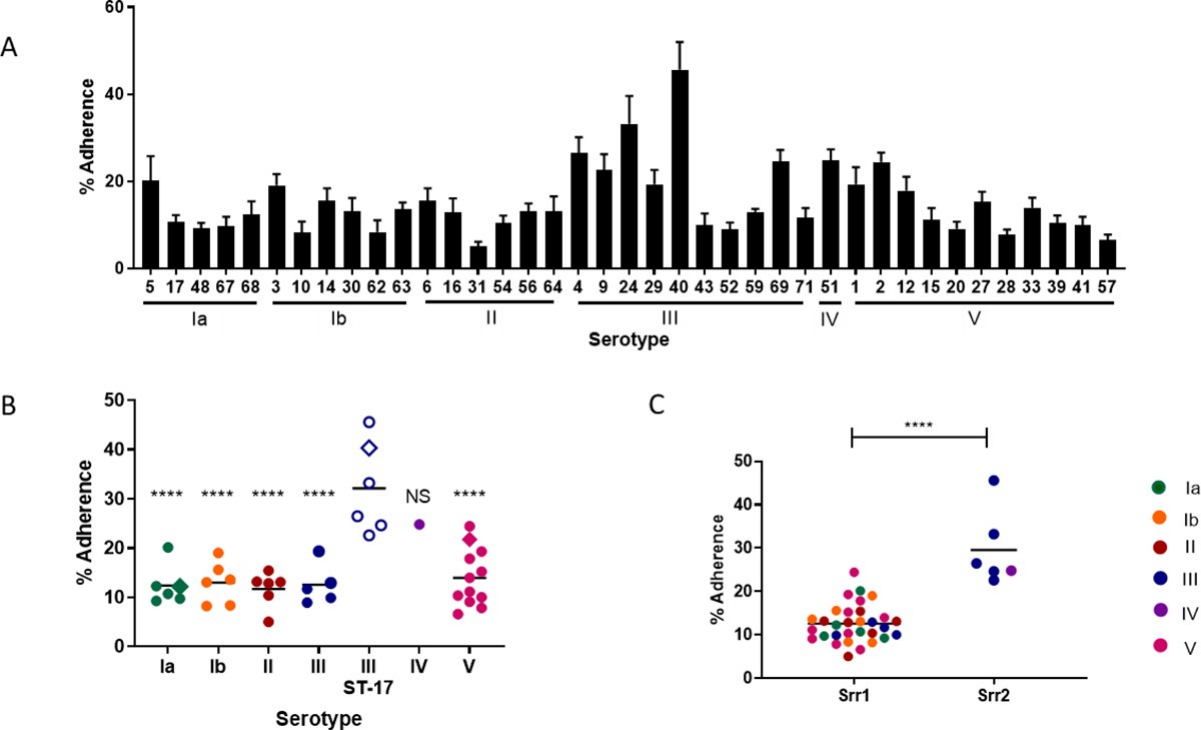
Vaginal epithelial cell adherence. A) Adherence of GBS clinical isolates to human vaginal epithelial cells (VK2/E6E7) at MOI = 1. Data represent the average of three independent experiments, each performed with three technical replicates. B) Adherence separated by serotype along the x-axis and compared to invasive isolates COH1, A909, and CJB111, denoted by diamonds. Open symbols represent isolates belonging to the hyper-virulent sequence type 17 (ST-17) lineage. Data were analyzed by a one-way ANOVA with Sidak’s multiple comparisons test, with each serotype compared to all others. Non-ST-17 samples (Ia, Ib, II, III, IV, and V) were compared to serotype III, ST-17 strains, **** *p* < 0.0001. No significant differences were detected between non-ST-17 samples. C) Adherence percentages separated by the presence of serine-rich repeat glycoproteins (Srr1 or Srr2). Symbol colors in panels B & C indicate capsular serotype. Data were analyzed by a one-way ANOVA with Sidak’s multiple comparisons test, **** *p* < 0.0001.

We have shown previously that GBS disease isolates are capable of adhering to human vaginal epithelial cells [17, 19, 39, 40]. To determine the ability of these colonizing isolates to adhere to vaginal epithelium, all strains were incubated with the human vaginal epithelial cell line VK2/E6E7 and adherent bacteria were recovered as described in the Methods. GBS vaginal isolates revealed a wide range of adherence (5–45% of the original inoculum, Fig 1A), levels similar to those of disease isolates of the same serotype: A909 (Ia), COH1 (III), and CJB111 (V) (denoted by the diamond symbols, Fig 1B).

Because ST-17 strains are commonly associated with invasive disease we assessed whether isolates of this strain background would exhibit increased attachment to VK2/E6E7 monolayers. Indeed, ST-17, serotype III strains adhered significantly better to vaginal epithelium compared to non-ST-17 serotype III strains (32.2% and 12.5%, respectively, *p* < 0.0001), suggesting that the ST-17 background confers an adherence advantage in serotype III strains (Fig 1B). Serotype III, ST-17 strains were significantly more adherent than serotypes Ia, Ib, II, and V (*p* < 0.0001), but exhibited similar adherence as the serotype IV strain as this strain also expressed Srr2 (Fig 1B). Additionally, no significant differences in adherence were detected across serotypes Ia, Ib, II, III non-ST-17, IV, and V (Fig 1B). As expected, we observed that Srr2*-*positive strains (which are typically of ST-17 lineage [44, 45]) exhibited increased adherence compared to Srr1*-*positive isolates (*p* < 0.0001) (Fig 1C).

### Antibiotic sensitivity profiling of clinical isolate panel

**Fig 2:**
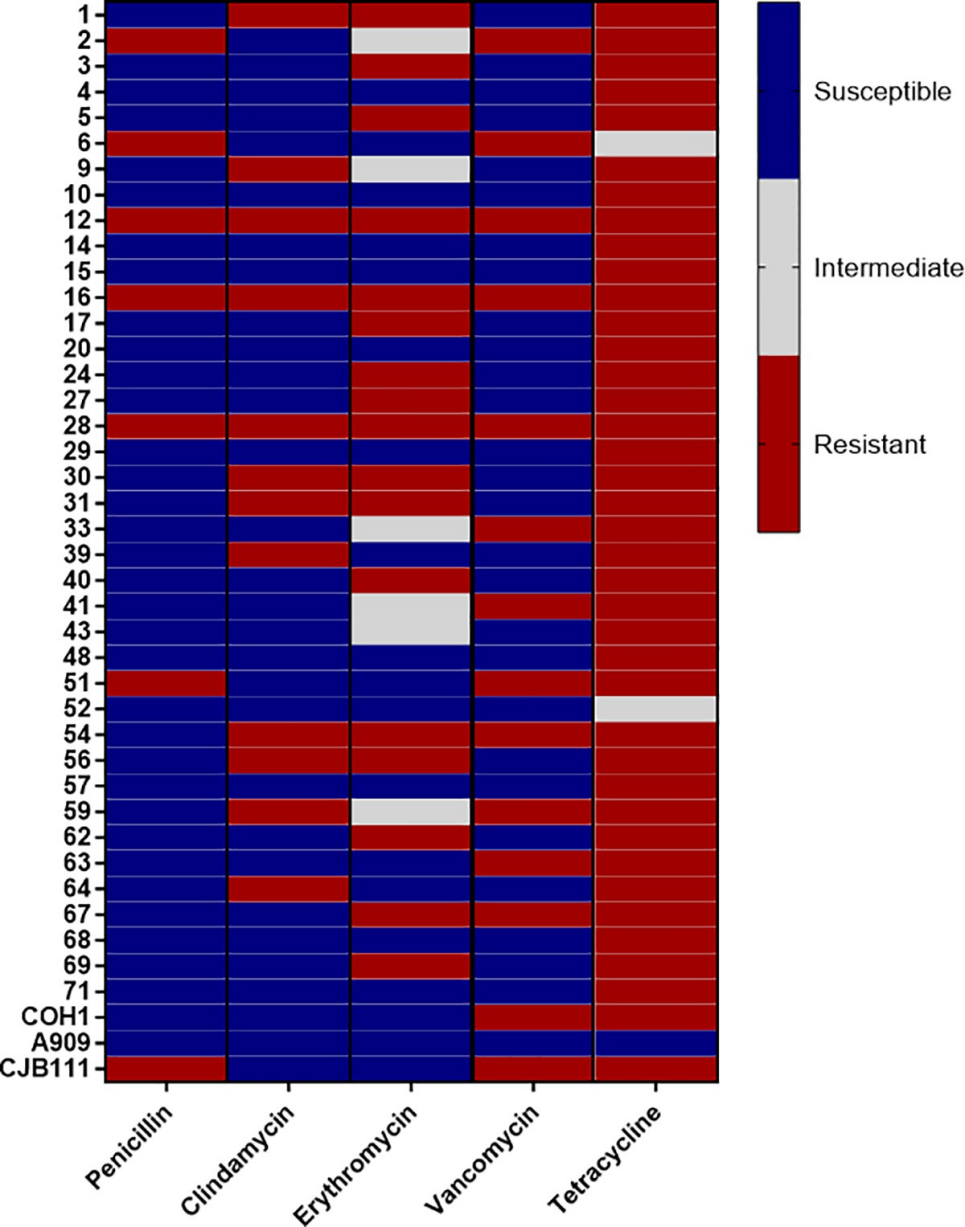
Antibiotic sensitivity of isolate panel. Antibiotic suscpetibility profiles (determined by disc diffusion measurement) displayed in a heat map with blue boxes indicating susceptible isolates, gray boxes indicating intermediate isolates, and red boxes indicating resistant isolates. Antibiotic discs were used at the following concentrations: penicillin 10 U, clindamycin 2 mg, erythromycin 15 mg, vancomycin 30 μg, and tetracycline 30 μg.

Rising antibiotic resistance has been reported in the literature in pathogens and commensal microbiota. As antibiotic prophylaxis during delivery is the primary measure taken to prevent GBS ascending infection and transmission to the neonate, it is imperative to monitor effective antibiotic treatment regimens. Currently, penicillin is the first-line antibiotic for pregnant women testing positive for GBS by rectovaginal swabs. In the case of penicillin allergy, women are prescribed clindamycin, erythromycin, or, vancomycin [1]. To determine the efficacy of these clinically relevant antibiotics against this panel of colonizing isolates, we used an antibiotic disc diffusion assay. Of the 39 isolates in this panel, 15.4% (6/39) were resistant to the first-line antibiotic, penicillin, 30.8% (12/39) were resistant to clindamycin, and 30.8% (12/39) were resistant to vancomycin (Table 4, Fig 2). Further, 59% (23/39) of isolates exhibited either resistance (17/39) or intermediate susceptibility (6/39) to erythromycin. Lastly, 94.9% (37/39) of strains were identified as tetracycline resistant with only 5% (2/39) displaying an intermediate resistance phenotype. Collectively all 39 strains were not susceptible to tetracycline; however, this is unsurprising as high tetracycline resistance in GBS has been observed historically [46]. To determine if capsular serotype is associated with GBS susceptibility to antibiotics, vaginal isolates were grouped by serotype (Table 4) and analyzed using Fisher’s exact test. We observed that resistance to penicillin and clindamycin were significantly associated with serotype (*p* = 0.03 and *p* = 0.04, respectively), with the majority of penicillin- and clindamycin-resistant strains belonging to serotypes II and V.

**Table 4:** Antibiotic sensitivity distribution by serotype.

	Antibiotic	Susceptible	Intermediate	Resistant
**Composite** **N = 39**	Penicillin	33 (84.6%)	NA	6 (15.4%)
	Clindamycin	27 (69.2%)	0	12 (30.8%)
	Erythromycin	16 (41.0%)	6 (15.4%)	17 (43.6%)
	Vancomycin	27 (69.2%)	NA	12 (30.8%)
	Tetracycline	0	2 (5.1%)	37 (94.9%)
**Serotype Ia** **N = 5**	Penicillin	5 (100%)	NA	0
	Clindamycin	5 (100%)	0	0
	Erythromycin	2 (40%)	0	3 (60%)
	Vancomycin	4 (80%)	NA	1 (20%)
	Tetracycline	0	0	5 (100%)
**Serotype Ib** **N = 6**	Penicillin	6 (100%)	NA	0
	Clindamycin	5 (83%)	0	1 (17%)
	Erythromycin	3 (50%)	0	3 (50%)
	Vancomycin	5 (83%)	NA	1 (17%)
	Tetracycline	0	0	6 (100%)
**Serotype II** **N = 6**	Penicillin	4 (67%)	NA	2 (33%)
	Clindamycin	1 (17%)	0	5 (83%)
	Erythromycin	2 (33%)	0	4 (67%)
	Vancomycin	3 (50%)	NA	3 (50%)
	Tetracycline	0	1 (17%)	5 (83%)
**Serotype III** **N = 10**	Penicillin	10 (100%)	NA	0
	Clindamycin	8 (80%)	0	2 (20%)
	Erythromycin	4 (40%)	3 (30%)	3 (30%)
	Vancomycin	9 (90%)	NA	1 (10%)
	Tetracycline	0	1 (10%)	9 (90%)
**Serotype IV** **N = 1**	Penicillin	0	NA	1 (100%)
	Clindamycin	1 (100%)	0	0
	Erythromycin	1 (100%)	0	0
	Vancomycin	0	NA	1 (100%)
	Tetracycline	0	0	1 (100%)
**Serotype V** **N = 11**	Penicillin	8 (73%)	NA	3 (27%)
	Clindamycin	7 (64%)	0	4 (36%)
	Erythromycin	4 (36%)	3 (27%)	4 (36%)
	Vancomycin	6 (55%)	NA	5 (45%)
	Tetracycline	0	0	11 (100%)

## Discussion

Herein we have characterized 39 GBS isolates obtained from the vaginal tracts of pregnant women. We have evaluated the serotype and antibiotic susceptibility of these isolates to front-line drugs and have functionally assessed isolates for parameters important for vaginal colonization, including adherence to human vaginal epithelial cells and hemolytic activity. We found that the most abundant serotypes in this collection were serotype V and III, with the order of most abundant to least abundant serotype being V > III > II > Ia/Ib > IV. The serotype distribution of our panel supports previous literature as either serotype V or III have been reported as the most prevalent serotype in 18 of 23 studies conducted on GBS colonizing isolates since 2007 (Table 5). Serotypes Ia, Ib, and II have also been reported among the top three most prevalent serotypes in these studies (Table 5). Serotype IV appears to be relatively rare as it was seldom seen in vaginal isolates across all studies, including the isolate cohort presented here. Although some studies of clinical isolates from Africa or Asia reported increased prevalence of more recently discovered serotypes VI, VII, and VIII, we did not identify any of these serotypes in our cohort.

**Table 5:** Serotype and antibiotic resistance profiles across relevant literature.

	Percentages of Antibiotic Resistance, n (%)						
Reference	Location	Prevalent Serotype	Penicillin 10 U	Vancomycin 30 μg	Erythromycin 15 μg	Clindamycin 2 μg	Tetracycline 30 μg
*Present Study*	Michigan, USA	V, 11/39 (28.2)	6/39 (15)	12/39 (31)	17/39 (44)	12/39 (31)	37/39 (95)
*A’Hearn-Thomas [47]*	Botswana	V, 24/53 (45.3)	___	___	___	___	___
*Belard et al*. *2015* [48]	Gabon, Africa	V, 33/109 (30.3)	0/109 (0)	___	0/109 (0)	0/109 (0)	___
*Botelho et al*. *2018* [49]	Brazil	Ia, 257/689 (37.3)	___	0/689 (0)	97/689 (14)	14/689 (2)	592/689 (86)
*Chukwu et al*. *2015* [50]	South Africa	III, 38/128 (29.7)	___	___	___	___	___
*Correa et al*. *2011* [51]	Brazil	Ia, 20/60 (33.2)	0/60 (0)	0/60 (0)	8/60 (13)	10/60 (17)%	49/60 (82)
*Dutra et al*. *2018* [52]	Brazil	Ia, 120/434 (27.6)	___	___	18/434 (4)	13/434 (3)	420/434 (97)
*El Aila et al*. *2009* [53]	Belgium	III (20.8)	___	___	11/40 (28)	3/40 (8)	___
*Eskandarian et al*. *2015* [54]	Malaysia	VI, 23/103 (22.3)	0/103 (0)	___	24/103 (23)	18/103 (18)	74/103 (72)
*Florindo et al*. *2010* [55]	Portugal	III, 35/100 (35)	0/100 (0)	0/100 (0)	19/100 (19)	______	___
*Hannoun et al*. *2009* [56]	Lebanon	III, 15/76 (19.7)	0/76 (0)	___	12/76 (16)	9/76 (12)	66/76 (87)
*Ji et al. 2017 [57]*	China	III, 84/153 (54.9)	0/153 (0)	0/153 (0)	99/153 (65)	80/153 (52)	___
*Khatami 2019 [58]*	New York, USA	III, 12/25 (48)	___	___	___	___	___
*Khodaei et al*. *2018* [59]	Iran	III, 62/90 (68.9)	0/90 (0)	1/90 (1)	27/90 (30)	89/90 (99)	89/90 (99)
*Liakopolous et al*. 2014 [60]	Greece	III, 17/26 (65.4)	0/26 (0)	0/26 (0)	22/26 (84.6)	21/26 (82)	26/26 (100)
*Mavenyengwa et al*. 2010 [61]	Zimbabwe	III, 47/121 (38.8)	___	___	___	___	___
*Pinto et al*. *2018* [62]	Portugal	III, 15/67 (22.4)	___	___	___	___	___
*Sadeh et al*. *2016* [63]	Iran	III, 50/100 (50)	___	___	___	___	___
*Seo et al. 2010 [64]*	Korea	III, 112/376 (29.8)	___	___	___	___	___
*Slotved et al*. *2017* [65]	Ghana	VII, 44/108 (40.7)	___	___	___	___	___
*Smith et al*. *2007* [66]	Michigan & Texas, USA	Ia, 101/421 (24)	___	___	___	___	___
*Teatero et al. 2017 [67]*	USA & Canada	III, 26/102 (25.5)	0/102 (0)	0/102 (0)	37/102 (36)	25/102 (25)	91/102 (89)
*Ueno et al*. *2012* [68]	Japan	V, 72/376 (19.1)	0/376 (0)	0/376 (0)	48/376 (13)	34/376 (9)	175/376 (47)
*Usein et al*. *2014* [69]	Bucharest	V, 20/55 (36.4)	0/43 (0)	___	19/43 (44)	19/43 (44)	43/43 (100)

In addition to serotyping, we functionally assessed our panel of isolates for hemolytic activity and the ability to adhere to human vaginal epithelial cells. When plated on blood agar, low levels of hemolysis (similar to that of COH1 and A909 strains) were detected in 35/39 vaginal isolates. Although there were some isolates that exhibited either non-detectable levels of hemolysis (similar to the COH1Δ*cylE* strain) (2/39) or high levels of hemolysis (similar to the hyper-hemolytic strain CNCTC10/84) (2/39) (Table 3). Previous studies have shown that GBS strains producing the β-H/C have an advantage in vaginal colonization as well as placental invasion compared to strains deficient in β-H/C [70, 71]. Since the majority of the vaginal isolates in our panel exhibit low levels of hemolytic activity, our data suggest that strains producing high levels of β-H/C may be selected against during colonization of the vagina. This is supported by our previous observations that hyper-hemolytic strain CNCTC10/84 resulted in increased pro-inflammatory cytokine production in vaginal epithelial cells and more rapid clearance from the murine vaginal tract [72]. The global regulator CovR regulates β-H/C, specifically repressing its expression in acidic environments, such as that of the vaginal mucosa [73, 74]. We therefore hypothesize that during vaginal carriage, CovR may suppress β-H/C expression resulting in decreased inflammation and more persistent GBS colonization. Alternatively, in neutral environments such as the chorioamniotic membrane, β-H/C may be expressed, potentiating invasion by hyper-hemolytic GBS strains [37].

Adherence to epithelial barriers promotes successful GBS colonization the vaginal tract. All strains in this cohort were able to adhere to vaginal epithelial cells, though adherence capabilities varied greatly across strains (Fig 1A). We observed that 5–45% of the original bacterial inoculum attached to VK2/E6E7 monolayers. Serotype III isolates exhibited the highest level of adherence compared to the isolates of the remaining serotypes tested. Of the serotype III isolates, ST-17 and Srr2 were associated with increased adherence compared to non-ST-17 strains, which typically express Srr1 (Fig 1B & 1C). Strains belonging to the hyper-virulent ST-17 background exclusively express serotype III capsule [75, 76] and are significantly associated with LoD and meningitis [75, 77, 78]. The propensity of ST-17/serotype III strains to cause invasive meningeal disease has been attributed to expression of ST-17 associated factors, such as hyper-virulent GBS adhesin (HvgA) and serine-rich repeat (Srr) glycoprotein Srr2 [11, 15, 38, 43, 76]. HvgA, a cell-wall anchored surface protein, has been implicated in colonization and invasion of both the intestine and the blood brain barrier, and detection of the *hvgA* gene (*gbs2018C*) is the basis for the ST-17 PCR used in this study and others [38]. The contribution of HvgA to GBS colonization of the female reproductive tract has not been demonstrated, but HvgA expression may contribute to the increased adherence by ST17/serotype III isolates to vaginal epithelium compared to other isolates observed in this study (Fig 1B). In addition to HvgA, ST-17 strains exclusively express Srr2 (instead of Srr1), which has been shown to promote virulence *in vivo* [15] and adherence to vaginal epithelium, *in vitro* [17]. Indeed, in the present study strains harboring the *srr2* gene exhibited increased adherence to vaginal epithelium compared to other isolates (Fig 1C). Previous studies have also shown that both Srr1 and Srr2 interact with host fibrinogen [18, 19, 43]; however, Srr2 specifically is more abundant on the bacterial cell surface and binds fibrinogen with an increased affinity compared to Srr1 [42, 43]. We therefore hypothesize that the increased epithelial adherence potential of these Srr2-expressing strains may promote increased transmission to the neonate and subsequently contribute to the development of neonatal infections [10, 79]. Previous literature indicates that bacterial hemolytic activity may also contribute to GBS adherence to host cells; yet, this association remains controversial [40, 80]. Our data suggest that hemolytic activity did not impact the ability of GBS to adhere to vaginal epithelium (S3 Fig). This has similarly been reported by *Whidbey et al*. [37].

Upon assessment of antibiotic susceptibility, we found that this set of clinical isolates exhibited various levels of antibiotic resistance. Specifically, we observed that 30.8% of isolates were resistant to clindamycin, 43.6% to erythromycin, and 94.9% to tetracycline (Fig 2, Table 4). High percentages of tetracycline resistance have been detected in GBS clinical isolates and reported since 1975 [46]. Of specific interest within our data set was an increased resistance to penicillin in roughly 15% of strains in our panel. This was particularly striking when penicillin resistance was broken down by serotype. Serotype II and V strain groups harbored all penicillin resistant isolates, exhibiting 33% and 27% penicillin resistance, respectively. Though the first GBS isolates displaying reduced penicillin susceptibility were detected in sputum samples in 2008 [81], penicillin resistance was not reported in GBS vaginal isolates until 2014 by Crespo-Ortiz et al. [82]. More recently, reports have detected reduced sensitivity to β-lactam antibiotics in GBS isolates collected from pregnant women [83, 84]. Our study and others indicate that not only is penicillin-resistance among vaginal isolates rising, but it may be localized to particular serotypes [83, 84]. Because of this, future GBS serotyping may be integral to effectively treating GBS-positive pregnant women before delivery. Additionally, many isolates in this collection were found to be vancomycin-resistant (Table 4), which has not been commonly reported in previous studies of vaginal GBS isolates. Although vancomycin-resistant isolates were found in every serotype present in our cohort, vancomycin resistance was enriched in serotypes II and V (50% and 45% resistant, respectively). Additionally, three isolates in our panel were resistant to all antibiotics tested. This poses a significant public health risk as antibiotics are the only current therapeutic for GBS infection.

In conclusion, here we have characterized a GBS clinical isolate cohort collected from the vaginal tracts of pregnant women by means of molecular and capsular serotyping, antibiotic susceptibility profiling, and assessing the virulence potential. The serotype prevalence and resistance of erythromycin, tetracycline and clindamycin within our cohort match other epidemiological studies in the literature for colonizing GBS isolates. We describe an emergence of vancomycin-resistant and penicillin-resistant strains, especially within serotypes II and V and describe three isolates that are resistant to all of the front-line antibiotics for GBS. Finally, our study confirms the importance of GBS surface protein Srr2 and ST-17 in successful GBS adherence to the vaginal epithelium. This collection of isolates will be a useful tool in testing strain heterogeneity in models of GBS colonization.

## Supporting information

S1 Fig Serotyping of vaginal clinical isolates by flow cytometry using anti-capsule monoclonal antibodies. Histograms display binding by various anti-capsular monoclonal antibodies (identified by color: serotype Ia, green; serotype Ib, orange; serotype II, red; serotype III, blue). The y-axis indicates number of events normalized to the mode and the x-axis indicates fluorescent intensity. Isotype control monoclonal antibodies against non-cognate capsule were utilized as negative controls and are indicated by the light gray histograms. All serotype Ia, Ib, II, and III isolates designated by molecular means (PCR) were tested by FCSA from two independent cultures in two independent experiments.
(TIF)

S2 Fig Hemolytic activity of colonizing clinical isolate panel. Hemolytic zone of clearance is shown for vaginal clinical isolates and for reference strains COH1Δ*cylE* (-), COH1 (+), A909 (++), and CNCTC10/84 (+++) after inoculation onto sheep blood agar plates.
(TIF)

S3 Fig Correlation of hemolytic activity with vaginal epithelial cell adherence. Adherence of GBS clinical isolates to human vaginal epithelial cells (VK2/E6E7) is shown separated by hemolytic activity (score of -, +, ++, +++ as determined in Table 3) along the x-axis. Symbol colors indicate capsular serotype assigned in Table 2. Data were analyzed by a one-way ANOVA with Sidak's multiple comparisons test, NS = not significant.
(TIF)

## References

[1] Verani JR, McGee L, Schrag SJJM, Mortality Weekly Report RGfC, Recommendations, Reports. Prevention of perinatal group B streptococcal disease. 2010;59(RR10):1–32. 21088663

[2] Melin P. Neonatal group B streptococcal disease: from pathogenesis to preventive strategies. Clinical Microbiology and Infection. 2011;17(9):1294–303. 10.1111/j.1469-0691.2011.03576.x 21672083

[3] Doran KS, Nizet V. Molecular pathogenesis of neonatal group B streptococcal infection: no longer in its infancy. Molecular microbiology. 2004;54(1):23–31. 10.1111/j.1365-2958.2004.04266.x 15458402

[4] Baker C, Edwards M. Group B Streptococcal infections, p. 1091–1156 Infectious diseases of the fetus and newborn infant, 5th ed WB Saunders, Philadelphia, Pa 2001.

[5] Schrag S, Gorwitz RJ, Fultz-Butts K, Schuchat A. Prevention of perinatal group B streptococcal disease; revised guidelines from CDC. MMWR Recomm Rep. 2002;51(RR11):1–22. 12211284

[6] Phares CR, Lynfield R, Farley MM, Mohle-Boetani J, Harrison LH, Petit S, et al Epidemiology of invasive group B streptococcal disease in the United States, 1999–2005. Jama. 2008;299(17):2056–65. 10.1001/jama.299.17.2056 18460666

[7] CDC. Active Bacterial Core Surveillance Report, Emerging Infections Program Network, Group B Streptococcus, 2017. 2017.

[8] Armistead B, Oler E, Waldorf KA, Rajagopal L. The Double Life of Group B Streptococcus: Asymptomatic Colonizer and Potent Pathogen. Journal of molecular biology. 2019. 10.1016/j.jmb.2019.01.035 PMC6646060 30711542

[9] Edmond KM, Kortsalioudaki C, Scott S, Schrag SJ, Zaidi AK, Cousens S, et al Group B streptococcal disease in infants aged younger than 3 months: systematic review and meta-analysis. The Lancet. 2012;379(9815):547–56. 10.1016/S0140-6736(11)61651-6 22226047

[10] Jones N, Bohnsack JF, Takahashi S, Oliver KA, Chan M-S, Kunst F, et al Multilocus sequence typing system for group B streptococcus. Journal of clinical microbiology. 2003;41(6):2530–6. 10.1128/JCM.41.6.2530-2536.2003 12791877 PMC156480

[11] Tazi A, Disson O, Bellais S, Bouaboud A, Dmytruk N, Dramsi S, et al The surface protein HvgA mediates group B streptococcus hypervirulence and meningeal tropism in neonates. Journal of experimental medicine. 2010;207(11):2313–22. 10.1084/jem.20092594 20956545 PMC2964583

[12] Nizet V, Gibson RL, Chi EY, Framson PE, Hulse M, Rubens CEJI, et al Group B streptococcal beta-hemolysin expression is associated with injury of lung epithelial cells. 1996;64(9):3818–26. 8751934 10.1128/iai.64.9.3818-3826.1996 PMC174298

[13] Doran KS, Chang JC, Benoit VM, Eckmann L, Nizet VJTJoid. Group B streptococcal β-hemolysin/cytolysin promotes invasion of human lung epithelial cells and the release of interleukin-8. 2002;185(2):196–203. 10.1086/338475 11807693

[14] Doran KS, Liu GY, Nizet VJTJoci. Group B streptococcal β-hemolysin/cytolysin activates neutrophil signaling pathways in brain endothelium and contributes to development of meningitis. 2003;112(5):736–44. 10.1172/JCI17335 12952922 PMC182187

[15] Seifert KN, Adderson EE, Whiting AA, Bohnsack JF, Crowley PJ, Brady LJJM. A unique serine-rich repeat protein (Srr-2) and novel surface antigen (ε) associated with a virulent lineage of serotype III *Streptococcus agalactiae*. 2006;152(4):1029–40. 10.1099/mic.0.28516-0 16549667

[16] Samen U, Eikmanns BJ, Reinscheid DJ, Borges FJI, immunity. The surface protein Srr-1 of *Streptococcus agalactiae* binds human keratin 4 and promotes adherence to epithelial HEp-2 cells. 2007;75(11):5405–14. 10.1128/IAI.00717-07 PMC2168289 17709412

[17] Sheen TR, Jimenez A, Wang N-Y, Banerjee A, van Sorge NM, Doran KS. Serine-Rich Repeat Proteins and Pili Promote *Streptococcus agalactiae* Colonization of the Vaginal Tract. 2011;193(24):6834–42. 10.1128/JB.00094-11 21984789 PMC3232834

[18] Seo HS, Mu R, Kim BJ, Doran KS, Sullam PMJPp. Binding of glycoprotein Srr1 of *Streptococcus agalactiae* to fibrinogen promotes attachment to brain endothelium and the development of meningitis. 2012;8(10):e1002947 10.1371/journal.ppat.1002947 23055927 PMC3464228

[19] Wang N-Y, Patras KA, Seo HS, Cavaco CK, Rösler B, Neely MN, et al Group B streptococcal serine-rich repeat proteins promote interaction with fibrinogen and vaginal colonization. 2014;210(6):982–91. 10.1093/infdis/jiu151 24620021 PMC4192050

[20] Maisey HC, Hensler M, Nizet V, Doran KSJJob. Group B streptococcal pilus proteins contribute to adherence to and invasion of brain microvascular endothelial cells. 2007;189(4):1464–7. 10.1128/JB.01153-06 17041051 PMC1797338

[21] Maisey HC, Quach D, Hensler ME, Liu GY, Gallo RL, Nizet V, et al A group B streptococcal pilus protein promotes phagocyte resistance and systemic virulence. 2008;22(6):1715–24. 10.1096/fj.07-093963 18198218 PMC2721339

[22] Banerjee A, Kim BJ, Carmona EM, Cutting AS, Gurney MA, Carlos C, et al Bacterial Pili exploit integrin machinery to promote immune activation and efficient blood-brain barrier penetration. 2011;2:462 10.1038/ncomms1474 21897373 PMC3195231

[23] Lauer P, Rinaudo CD, Soriani M, Margarit I, Maione D, Rosini R, et al Genome analysis reveals pili in Group B Streptococcus. 2005;309(5731):105–. 10.1126/science.1111563 15994549

[24] Dramsi S, Caliot E, Bonne I, Guadagnini S, Prévost MC, Kojadinovic M, et al Assembly and role of pili in group B streptococci. 2006;60(6):1401–13. 10.1111/j.1365-2958.2006.05190.x 16796677

[25] Jacobsson KJVm. A novel family of fibrinogen-binding proteins in *Streptococcus agalactiae*. 2003;96(1):103–13. 10.1016/s0378-1135(03)00206-2 14516712

[26] Madoff LC, Michel JL, Kasper DL. A monoclonal antibody identifies a protective C-protein alpha-antigen epitope in group B streptococci. 1991;59(1):204–10. 1702759 10.1128/iai.59.1.204-210.1991 PMC257727

[27] Faralla C, Metruccio MM, De Chiara M, Mu R, Patras KA, Muzzi A, et al Analysis of Two-Component Systems in Group B Streptococcus Shows That RgfAC and the Novel FspSR Modulate Virulence and Bacterial Fitness. mBio. 2014;5(3):e00870–14. 10.1128/mBio.00870-14 24846378 PMC4030450

[28] Wilkinson HW. Nontypable group B streptococci isolated from human sources. 1977;6(2):183–4. 408376 10.1128/jcm.6.2.183-184.1977 PMC274732

[29] Wilson CB, Weaver WMJJoID. Comparative susceptibility of group B streptococci and *Staphylococcus aureus* to killing by oxygen metabolites. 1985;152(2):323–9. 10.1093/infdis/152.2.323 2993435

[30] Hayano S, Tanaka A. Repetitive counterelectrophoresis on agar gel for the immunological identification of esterases produced by strains of Lancefield's group A, B, and C streptococci. Infect Immun. 1977;15(1):295–9. 319062 10.1128/iai.15.1.295-299.1977 PMC421361

[31] Poyart C, Tazi A, Réglier-Poupet H, Billoët A, Tavares N, Raymond J, et al Multiplex PCR assay for rapid and accurate capsular typing of group B streptococci. Journal of clinical microbiology. 2007;45(6):1985–8. 10.1128/JCM.00159-07 17376884 PMC1933079

[32] Geno KA, Saad JS, Nahm MH. Discovery of Novel Pneumococcal Serotype 35D, a Natural WciG-Deficient Variant of Serotype 35B. J Clin Microbiol. 2017;55(5):1416–25. 10.1128/JCM.00054-17 28202800 PMC5405259

[33] Wharton RE, Stefanov EK, King RG, Kearney JF. Antibodies generated against Streptococci protect in a mouse model of disseminated aspergillosis. J Immunol. 2015;194(9):4387–96. 10.4049/jimmunol.1401940 25821219 PMC4402265

[34] Pritchard DG, Gray BM, Egan ML. Murine monoclonal antibodies to type Ib polysaccharide of group B streptococci bind to human milk oligosaccharides. Infection and immunity. 1992;60(4):1598–602. 1548081 10.1128/iai.60.4.1598-1602.1992 PMC257035

[35] Gray BM, Egan ML, Pritchard DGJPr. Specificity of monoclonal antibodies against group B streptococcus type II and inhibition of their binding by human secretions. 1988;24(1):68 10.1203/00006450-198807000-00017 2457865

[36] Egan ML, Pritchard DG, Dillon H, Gray BJJoEM. Protection of mice from experimental infection with type III Group B Streptococcus using monoclonal antibodies. 1983;158(3):1006–11. 10.1084/jem.158.3.1006 6193228 PMC2187106

[37] Whidbey C, Harrell MI, Burnside K, Ngo L, Becraft AK, Iyer LM, et al A hemolytic pigment of Group B Streptococcus allows bacterial penetration of human placenta. Journal of Experimental Medicine. 2013;210(6):1265–81. 10.1084/jem.20122753 23712433 PMC3674703

[38] Lamy M-C, Dramsi S, Billoët A, Réglier-Poupet H, Tazi A, Raymond J, et al Rapid detection of the “highly virulent” Group B Streptococcus ST-17 clone. Microbes and infection. 2006;8(7):1714–22. 10.1016/j.micinf.2006.02.008 16822689

[39] Patras KA, Rosler B, Thoman ML, Doran KS. Characterization of host immunity during persistent vaginal colonization by Group B Streptococcus. Mucosal immunology. 2015;8(6):1339–48. 10.1038/mi.2015.23 25850655 PMC4598252

[40] Patras KA, Wang NY, Fletcher EM, Cavaco CK, Jimenez A, Garg M, et al Group B Streptococcus CovR regulation modulates host immune signalling pathways to promote vaginal colonization. 2013;15(7):1154–67. 10.1111/cmi.12105 23298320 PMC3657335

[41] Biemer JJ. Antimicrobial susceptibility testing by the Kirby-Bauer disc diffusion method. Annals of Clinical & Laboratory Science. 1973;3(2):135–40. 4575155

[42] Seo HS, Minasov G, Seepersaud R, Doran KS, Dubrovska I, Shuvalova L, et al Characterization of fibrinogen binding by glycoproteins Srr1 and Srr2 of *Streptococcus agalactiae*. Journal of Biological Chemistry. 2013;288(50):35982–96. 10.1074/jbc.M113.513358 24165132 PMC3861647

[43] Six A, Bellais S, Bouaboud A, Fouet A, Gabriel C, Tazi A, et al Srr2, a multifaceted adhesin expressed by ST‐17 hypervirulent G roup B Streptococcus involved in binding to both fibrinogen and plasminogen. Molecular microbiology. 2015;97(6):1209–22. 10.1111/mmi.13097 26094503

[44] Seifert KN, Adderson EE, Whiting AA, Bohnsack JF, Crowley PJ, Brady LJ. A unique serine-rich repeat protein (Srr-2) and novel surface antigen (epsilon) associated with a virulent lineage of serotype III Streptococcus agalactiae. Microbiology. 2006;152(Pt 4):1029–40. 10.1099/mic.0.28516-0 16549667

[45] Six A, Bellais S, Bouaboud A, Fouet A, Gabriel C, Tazi A, et al Srr2, a multifaceted adhesin expressed by ST-17 hypervirulent Group B Streptococcus involved in binding to both fibrinogen and plasminogen. Mol Microbiol. 2015;97(6):1209–22. 10.1111/mmi.13097 26094503

[46] Anthony BF, Concepcion NF. Group B Streptococcus in a general hospital. J Infect Dis. 1975;132(5):561–7. 10.1093/infdis/132.5.561 1102616

[47] A'Hearn-Thomas B, Khatami A, Randis TM, Vurayai M, Mokomane M, Arscott-Mills T, et al High Rate of Serotype V *Streptococcus agalactiae* Carriage in Pregnant Women in Botswana. The American journal of tropical medicine and hygiene. 2019. 10.4269/ajtmh.18-0847 PMC6493924 30915949

[48] Belard S, Toepfner N, Capan-Melser M, Mombo-Ngoma G, Zoleko-Manego R, Groger M, et al *Streptococcus agalactiae* serotype distribution and antimicrobial susceptibility in pregnant women in Gabon, Central Africa. 2015;5:17281 10.1038/srep17281 26603208 PMC4658565

[49] Botelho ACN, Oliveira JG, Damasco AP, Santos KT, Ferreira AFM, Rocha GT, et al *Streptococcus agalactiae* carriage among pregnant women living in Rio de Janeiro, Brazil, over a period of eight years. 2018;13(5):e0196925 10.1371/journal.pone.0196925 29750801 PMC5947911

[50] Chukwu MO, Mavenyengwa RT, Monyama CM, Bolukaoto JY, Lebelo SL, Maloba MR, et al Antigenic distribution of *Streptococcus agalactiae* isolates from pregnant women at Garankuwa hospital–South Africa. 2015;5(4):125 10.11599/germs.2015.1080 26716101 PMC4691193

[51] Corrêa ABdA, Silva LGd, Pinto TdCA, Oliveira ICMd, Fernandes FG, Costa NSd, et al The genetic diversity and phenotypic characterisation of *Streptococcus agalactiae* isolates from Rio de Janeiro, Brazil. 2011;106(8):1002–6. 10.1590/s0074-02762011000800017 22241124

[52] Dutra VG, Alves VM, Olendzki AN, Dias CA, de Bastos AF, Santos GO, et al *Streptococcus agalactiae* in Brazil: serotype distribution, virulence determinants and antimicrobial susceptibility. 2014;14(1):323. 10.1186/1471-2334-14-323 PMC4061772 24919844

[53] El Aila NA, Tency I, Claeys G, Saerens B, De Backer E, Temmerman M, et al Genotyping of *Streptococcus agalactiae* (group B streptococci) isolated from vaginal and rectal swabs of women at 35–37 weeks of pregnancy. 2009;9(1):153. 10.1186/1471-2334-9-153 PMC2753344 19747377

[54] Eskandarian N, Ismail Z, Neela V, Van Belkum A, Desa M, Nordin SAJEJoCM, et al Antimicrobial susceptibility profiles, serotype distribution and virulence determinants among invasive, non-invasive and colonizing *Streptococcus agalactiae* (group B streptococcus) from Malaysian patients. 2015;34(3):579–84. 10.1007/s10096-014-2265-x 25359580 PMC4356882

[55] Florindo C, Viegas S, Paulino A, Rodrigues E, Gomes JP, Borrego MJJCM, et al Molecular characterization and antimicrobial susceptibility profiles in *Streptococcus agalactiae* colonizing strains: association of erythromycin resistance with subtype III-1 genetic clone family. 2010;16(9):1458–63. 10.1111/j.1469-0691.2009.03106.x 19886900

[56] Hannoun A, Shehab M, Khairallah M-T, Sabra A, Abi-Rached R, Bazi T, et al Correlation between group B streptococcal genotypes, their antimicrobial resistance profiles, and virulence genes among pregnant women in Lebanon. 2010;2009. 10.1155/2009/796512 PMC2817894 20148175

[57] Ji W, Zhang L, Guo Z, Xie S, Yang W, Chen J, et al Colonization prevalence and antibiotic susceptibility of Group B Streptococcus in pregnant women over a 6-year period in Dongguan, China. PloS one. 2017;12(8):e0183083 10.1371/journal.pone.0183083 28813477 PMC5557540

[58] Khatami A, Randis TM, Tavares L, Gegick M, Suzman E, Ratner AJ. Vaginal co-colonization with multiple Group B Streptococcus serotypes. Vaccine. 2019;37(3):409–11. 10.1016/j.vaccine.2018.12.001 30528847 PMC6321774

[59] Khodaei F, Najafi M, Hasani A, Kalantar E, Sharifi E, Amini A, et al Pilus–encoding islets in *S*. *agalactiae* and its association with antibacterial resistance and serotype distribution. 2018;116:189–94. 10.1016/j.micpath.2018.01.035 29371153

[60] Liakopoulos A, Mavroidi A, Vourli S, Panopoulou M, Zachariadou L, Chatzipanagiotou S, et al Molecular characterization of *Streptococcus agalactiae* from vaginal colonization and neonatal infections: a 4-year multicenter study in Greece. 2014;78(4):487–90. 10.1016/j.diagmicrobio.2013.12.017 24503505

[61] Mavenyengwa R, Maeland J, Moyo SJIjomm. Serotype markers in a *Streptococcus agalactiae* strain collection from Zimbabwe. 2010;28(4):313 10.4103/0255-0857.71819 20966561

[62] Pinto AM, Pereira TA, Alves V, Araújo A, Lage OM. Incidence and serotype characterisation of *Streptococcus agalactiae* in a Portuguese hospital. 2018;71(6):508–13. 10.1136/jclinpath-2017-204646 29180508

[63] Sadeh M, Firouzi R, Derakhshandeh A, Khalili MB, Kong F, Kudinha TJJjom. Molecular characterization of *Streptococcus agalactiae* isolates from pregnant and non-pregnant women at Yazd University Hospital, Iran. 2016;9(2). 10.5812/jjm.30412 27127592 PMC4842249

[64] Seo YS, Srinivasan U, Oh KY, Shin JH, Chae JD, Kim MY, et al Changing molecular epidemiology of group B streptococcus in Korea. J Korean Med Sci. 2010;25(6):817–23. 10.3346/jkms.2010.25.6.817 20514299 PMC2877223

[65] Slotved H-C, Dayie NT, Banini JA, Frimodt-Møller NJBp, childbirth. Carriage and serotype distribution of *Streptococcus agalactiae* in third trimester pregnancy in southern Ghana. 2017;17(1):238 10.1186/s12884-017-1419-0 28732495 PMC5520380

[66] Smith T, Roehl S, Pillai P, Li S, Marrs C, Foxman BJE, et al Distribution of novel and previously investigated virulence genes in colonizing and invasive isolates of *Streptococcus agalactiae*. 2007;135(6):1046–54. 10.1017/S0950268806007515 17156495 PMC2870641

[67] Teatero S, Ferrieri P, Martin I, Demczuk W, McGeer A, Fittipaldi N. Serotype Distribution, Population Structure, and Antimicrobial Resistance of Group B Streptococcus Strains Recovered from Colonized Pregnant Women. J Clin Microbiol. 2017;55(2):412–22. 10.1128/JCM.01615-16 27852675 PMC5277510

[68] Ueno H, Yamamoto Y, Yamamichi A, Kikuchi K, Kobori S, Miyazaki MJJjoid. Characterization of group B streptococcus isolated from women in Saitama city, Japan. 2012;65(6):516–21. 10.7883/yoken.65.516 23183204

[69] Usein C-R, Militaru M, Cristea V, Străuţ MJMdIOC. Genetic diversity and antimicrobial resistance in *Streptococcus agalactiae* strains recovered from female carriers in the Bucharest area. 2014;109(2):189–96. 10.1590/0074-0276140431 24676662 PMC4015262

[70] Randis TM, Gelber SE, Hooven TA, Abellar RG, Akabas LH, Lewis EL, et al Group B Streptococcus β-hemolysin/cytolysin breaches maternal-fetal barriers to cause preterm birth and intrauterine fetal demise in vivo. The Journal of infectious diseases. 2014;210(2):265–73. 10.1093/infdis/jiu067 24474814 PMC4092248

[71] Carey AJ, Tan CK, Mirza S, Irving-Rodgers H, Webb RI, Lam A, et al Infection and cellular defense dynamics in a novel 17β-estradiol murine model of chronic human group B streptococcus genital tract colonization reveal a role for hemolysin in persistence and neutrophil accumulation. The Journal of Immunology. 2014;192(4):1718–31. 10.4049/jimmunol.1202811 24453257

[72] Patras KA, Doran KS. A murine model of group B Streptococcus vaginal colonization. JoVE (Journal of Visualized Experiments). 2016(117):e54708. 10.3791/54708 PMC5226234 27911391

[73] Jiang S-M, Cieslewicz MJ, Kasper DL, Wessels MR. Regulation of virulence by a two-component system in group B streptococcus. Journal of Bacteriology. 2005;187(3):1105–13. 10.1128/JB.187.3.1105-1113.2005 15659687 PMC545708

[74] Park SE, Jiang S, Wessels MR. CsrRS and environmental pH regulate group B streptococcus adherence to human epithelial cells and extracellular matrix. Infection and immunity. 2012;80(11):3975–84. 10.1128/IAI.00699-12 22949550 PMC3486057

[75] Jones N, Oliver KA, Barry J, Harding RM, Bisharat N, Spratt BG, et al Enhanced invasiveness of bovine-derived neonatal sequence type 17 group B streptococcus is independent of capsular serotype. Clin Infect Dis. 2006;42(7):915–24. 10.1086/500324 16511753

[76] Brochet M, Couve E, Zouine M, Vallaeys T, Rusniok C, Lamy MC, et al Genomic diversity and evolution within the species *Streptococcus agalactiae*. Microbes Infect. 2006;8(5):1227–43. 10.1016/j.micinf.2005.11.010 16529966

[77] Manning SD, Lewis MA, Springman AC, Lehotzky E, Whittam TS, Davies HD. Genotypic diversity and serotype distribution of group B streptococcus isolated from women before and after delivery. Clin Infect Dis. 2008;46(12):1829–37. 10.1086/588296 18462173 PMC9491394

[78] Manning SD, Springman AC, Lehotzky E, Lewis MA, Whittam TS, Davies HD. Multilocus sequence types associated with neonatal group B streptococcal sepsis and meningitis in Canada. J Clin Microbiol. 2009;47(4):1143–8. 10.1128/JCM.01424-08 19158264 PMC2668308

[79] Takahashi S, Adderson EE, Nagano Y, Nagano N, Briesacher MR, Bohnsack JF. Identification of a Highly Encapsulated, Genetically Related Group of Invasive Type III Group B Streptococci. The Journal of Infectious Diseases. 1998;177(4):1116–9. 10.1086/517408 9534996

[80] Lembo A, Gurney MA, Burnside K, Banerjee A, De Los Reyes M, Connelly JE, et al Regulation of CovR expression in Group B Streptococcus impacts blood–brain barrier penetration. Molecular microbiology. 2010;77(2):431–43. 10.1111/j.1365-2958.2010.07215.x 20497331 PMC2909351

[81] Kimura K, Suzuki S, Wachino J-i, Kurokawa H, Yamane K, Shibata N, et al First molecular characterization of group B streptococci with reduced penicillin susceptibility. 2008;52(8):2890–7. 10.1128/AAC.00185-08 18490507 PMC2493108

[82] Crespo-Ortiz MdP, Castañeda-Ramirez CR, Recalde-Bolaños M, Vélez-Londoño JDJBID. Emerging trends in invasive and noninvasive isolates of *Streptococcus agalactiae* in a Latin American hospital: a 17-year study. 2014;14(1):428. 10.1186/1471-2334-14-428 PMC4131052 25086463

[83] Moroi H, Kimura K, Kotani T, Tsuda H, Banno H, Jin W, et al Isolation of Group B Streptococcus with reduced β-lactam susceptibility from pregnant women. 2019;8(1):2–7. 10.1080/22221751.2018.1557987 30866792 PMC6455180

[84] Gizachew M, Tiruneh M, Moges F, Tessema BJAocm, antimicrobials. *Streptococcus agalactiae* maternal colonization, antibiotic resistance and serotype profiles in Africa: a meta-analysis. 2019;18(1):14. 10.1186/s12941-019-0313-1 PMC6437888 30922308

