# Supplementary figures and images for: Determinants of Group B streptococcal virulence potential amongst vaginal clinical isolates from pregnant women
Source: PLoS One. 2019 Dec 18;14(12):e0226699. doi: 10.1371/journal.pone.0226699 (PMC6919605; doi:10.1371/journal.pone.0226699)

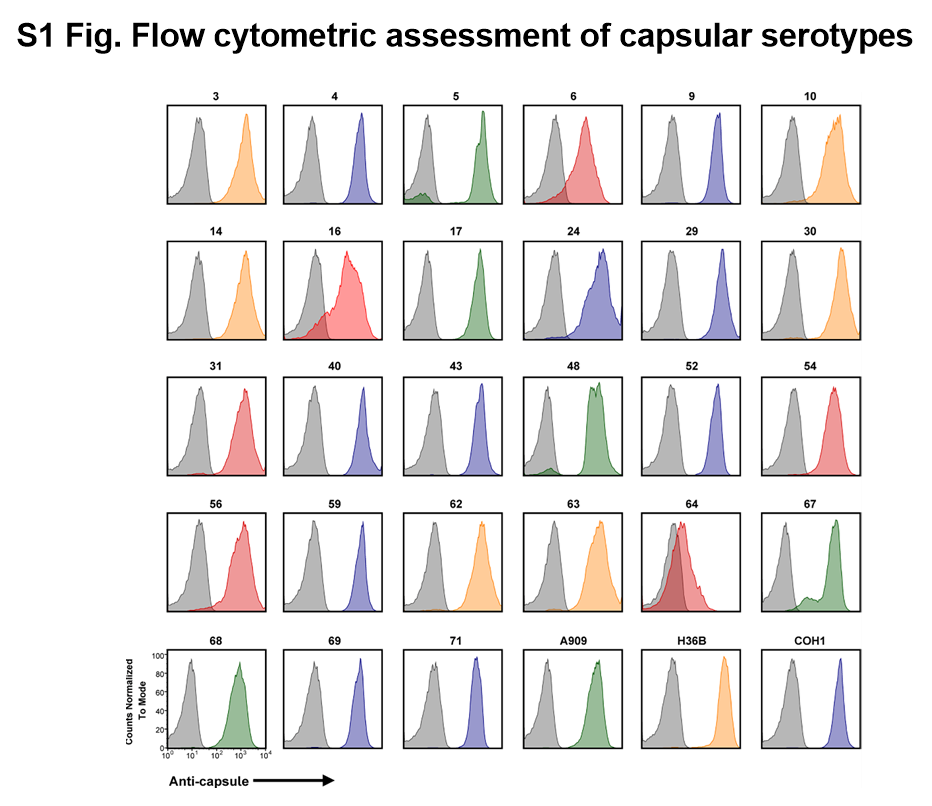

Supplement: S1 Fig — Histograms display binding by various anti-capsular monoclonal antibodies (identified by color: serotype Ia, green; serotype Ib, orange; serotype II, red; serotype III, blue). The y-axis indicates number of events normalized to the mode and the x-axis indicates fluorescent intensity. Isotype control monoclonal antibodies against non-cognate capsule were utilized as negative controls and are indicated by the light gray histograms. All serotype Ia, Ib, II, and III isolates designated by molecular means (PCR) were tested by FCSA from two independent cultures in two independent experiments. (TIF) [file pone.0226699.s001.tif]

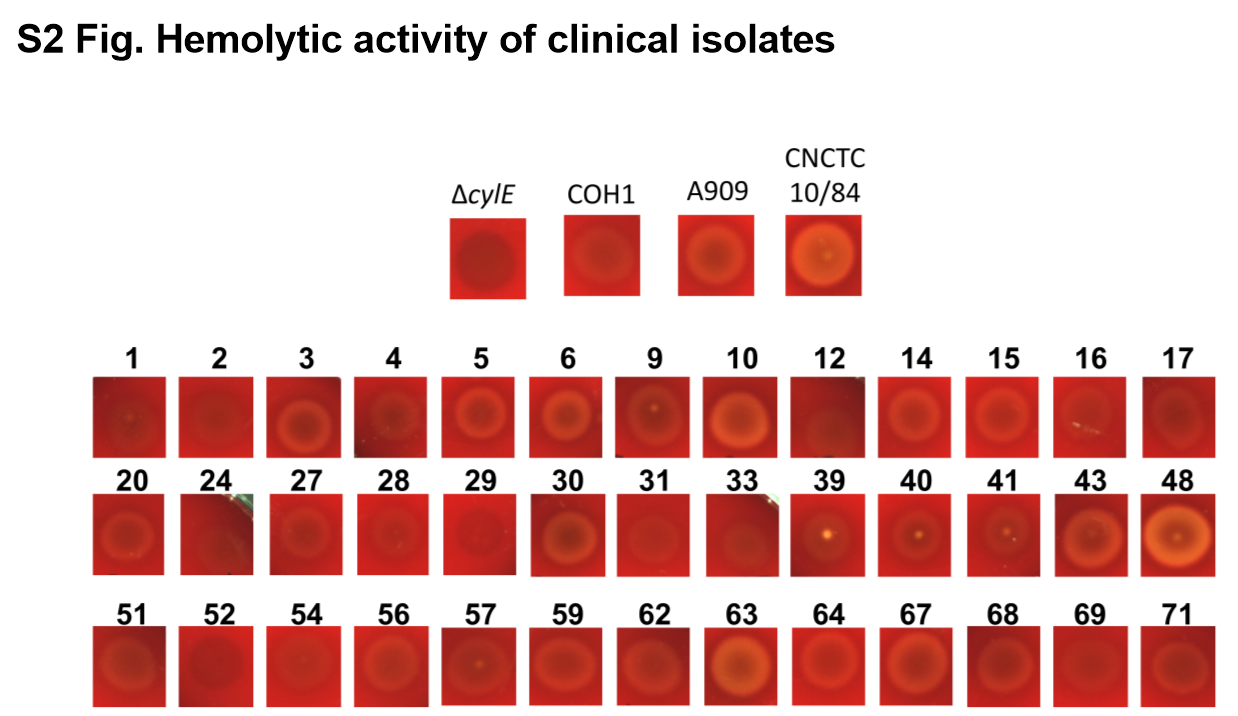

Supplement: S2 Fig — Hemolytic zone of clearance is shown for vaginal clinical isolates and for reference strains COH1ΔcylE (-), COH1 (+), A909 (++), and CNCTC10/84 (+++) after inoculation onto sheep blood agar plates. (TIF) [file pone.0226699.s002.tif]

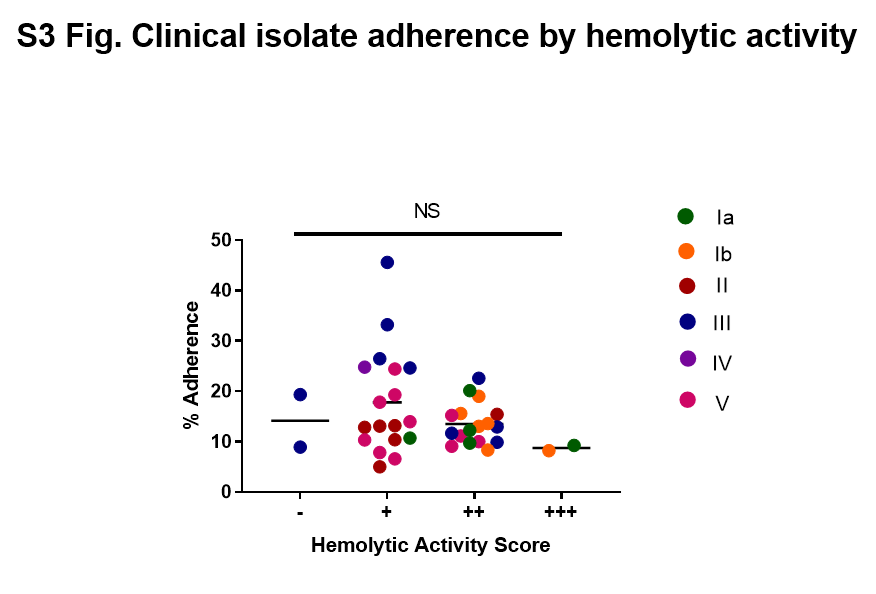

Supplement: S3 Fig — Adherence of GBS clinical isolates to human vaginal epithelial cells (VK2/E6E7) is shown separated by hemolytic activity (score of -, +, ++, +++ as determined in Table 3) along the x-axis. Symbol colors indicate capsular serotype assigned in Table 2. Data were analyzed by a one-way ANOVA with Sidak's multiple comparisons test, NS = not significant. (TIF) [file pone.0226699.s003.tif]
